# Ocular Hypertension and Glaucoma in Graves’ Orbitopathy: A Single-Center Retrospective Cross-Sectional Study

**DOI:** 10.3390/jcm15197741

**Published:** 2026-10-08

**Authors:** Alexandra Magdalena Ioana, Cătălin Prodan-Bărbulescu, Alina Maria Şişu, Sorin Lucian Bolintineanu

**Affiliations:** 1 Department I, Discipline of Anatomy and Embriology, “Victor Babes” University of Medicine and Pharmacy, 300041 Timisoara, Romania; alexandra.ioana@umft.ro (A.M.I.); alinasisu@umft.ro (A.M.Ş.); s.bolintineanu@umft.ro (S.L.B.); 2 Doctoral School, “Victor Babes” University of Medicine and Pharmacy Timisoara, Eftimie Murgu Square 2, 300041 Timisoara, Romania; 3 2nd Surgery Clinic, Timisoara Emergency County Hospital, 300723 Timisoara, Romania

**Keywords:** Graves’ orbitopathy, intraocular pressure, glaucoma, optical coherence tomography, ganglion cell layer, visual field, structure-function correlation

## Abstract

**Background/Objectives:** Elevated intraocular pressure (IOP) in Graves’ orbitopathy (GO) is a recognized risk factor for glaucomatous optic neuropathy, but the extent to which it translates into measurable structural and functional damage remains unclear. This study investigated structural and functional correlations in patients with GO stratified by IOP and glaucoma status. **Methods:** In this retrospective, cross-sectional study, 165 adult patients with GO were stratified into three groups of 55 each: normal IOP, ocular hypertension, and clinically diagnosed glaucoma. All patients underwent clinical examination, optical coherence tomography (OCT), and, where available, standard automated perimetry. Structural and functional parameters were compared across all three groups, with post-hoc pairwise testing. Multivariable logistic regression (clinical and combined models, adjusted for smoking and diabetes mellitus, restricted to the ocular hypertension and glaucoma groups) was used to identify factors independently associated with glaucoma status, with model discrimination assessed by optimism-corrected receiver operating characteristic (ROC) curve analysis. **Results:** IOP increased and best-corrected visual acuity (BCVA) declined progressively across groups. Most structural and functional parameters differentiated the glaucoma group from both other groups but did not distinguish normal IOP from ocular hypertension. In multivariable analysis restricted to the ocular hypertension and glaucoma groups, IOP was the factor most strongly associated with glaucoma status (odds ratio [OR] 1.44 in the clinical model and 1.61 after adjustment for structural parameters, both *p* ≤ 0.003), with BCVA also independently associated in the clinical model (*p* = 0.032). Diabetes mellitus and smoking were independently associated with glaucoma status in both models; the inverse association observed for smoking lacks an established biological explanation. Adding ganglion cell layer plus inner plexiform layer (GCL+IPL) thickness improved model discrimination (optimism-corrected area under the curve from 0.73 to 0.76) and was independently associated with glaucoma (OR 0.90, *p* = 0.036). **Conclusions:** IOP remains the factor most consistently and independently associated with glaucoma status in GO, with GCL+IPL thickness providing an additional independent contribution once smoking and diabetes mellitus are accounted for. These findings support regular IOP and visual acuity monitoring as first-line surveillance in GO, with structural OCT as a useful adjunct.

## 1. Introduction

Graves’ orbitopathy (GO), also known as thyroid eye disease, is an autoimmune inflammatory disorder characterized by orbital tissue expansion, extraocular muscle enlargement, and increased orbital pressure. Affecting nearly 40% of patients with Graves’ disease (GD), it constitutes the most frequent extrathyroidal manifestation of the disease [1,2]. GO is driven by thyrotropin receptor antibody (TRAb)-mediated activation of orbital fibroblasts expressing TSH receptors, which triggers an inflammatory cascade with cytokine release, glycosaminoglycan accumulation, and subsequent orbital tissue expansion and increased intraorbital pressure [3]. Severe forms of GO are characterized by marked facial disfiguration (exophthalmos, staring appearance), inflammatory manifestations, ocular dysfunction including diplopia, and, less frequently, vision loss due to compressive dysthyroid optic neuropathy (DON) [4,5]. The significant changes in facial appearance and functional impairment associated with the disease, along with its adverse impact on quality of life, psychological well-being, and socioeconomic status, are well recognized [6,7].

Since its initial description by Wessely in 1918 [8], elevated intraocular pressure (IOP) in GO has been examined in numerous studies focusing on its occurrence, clinical features and management [9,10,11]. Elevated IOP in GO represents a significant clinical finding that is not included in current disease severity classifications. Although relatively infrequent, GO-associated IOP elevation is an important risk factor for the development of glaucomatous optic neuropathy and may ultimately lead to optic nerve damage [10]. Elevated IOP in GO is multifactorial and may result from increased episcleral venous pressure, orbital tissue crowding, and mechanical restriction of extraocular muscles and lead to several anterior and posterior segment alterations [9,12,13,14]. Unlike primary open-angle glaucoma, where optic neuropathy develops primarily due to intrinsic susceptibility of the optic nerve head [15], IOP elevation in GO occurs within a distinct pathophysiological context. These differences raise important questions regarding the nature and extent of optic nerve damage in GO patients with increased IOP and whether such changes follow patterns comparable to glaucomatous optic neuropathy [16].

Optical coherence tomography (OCT) is an advanced imaging technique that uses high-resolution visualization of retinal microstructure [12]. Furthermore, the integration of OCT and optical coherence tomography angiography (OCTA) parameters may support clinical decision-making and guide treatment strategies across different stages of GO [17,18]. In parallel, standard automated perimetry allows functional assessment of visual field sensitivity, facilitating the investigation of structure–function relationships in GO [19]. While such correlations are well established in primary open-angle glaucoma [20], their behavior in GO-associated ocular hypertension remains insufficiently explored.

Several studies have reported increased IOP in patients with GO [21,22]; however, the extent to which this translates into measurable structural and functional damage remains unclear. IOP elevation does not appear to be a uniform feature of GO itself [12]. Therefore, the present study aims to investigate the correlation between structural and functional changes in patients with Graves’ orbitopathy, stratified according to intraocular pressure status and the presence of glaucomatous damage. By comparing patients with GO and normal IOP, GO with ocular hypertension, and GO with clinically diagnosed glaucomatous optic neuropathy, this study seeks to elucidate the relationship between OCT-derived structural parameters and visual field outcomes. Understanding these relationships may improve the early identification of optic nerve involvement in GO.

## 2. Materials and Methods

### 2.1. Study Design and Participants

**Figure 1:**
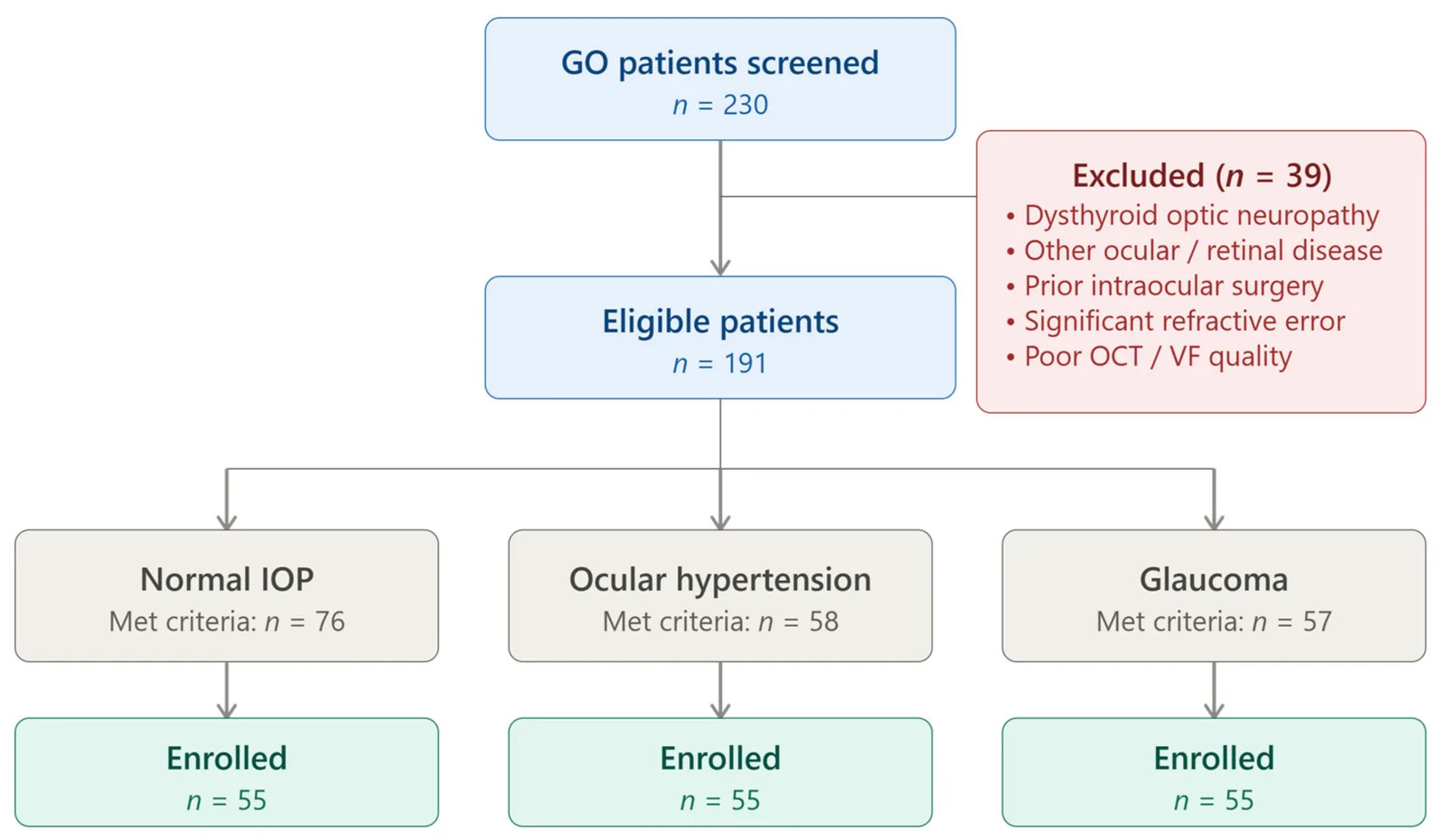
Participant flow diagram.

This was a retrospective cross-sectional l study conducted at the County Emergency Clinical Hospital “Pius Brînzeu” Timișoara, between January 2023 and December 2025. Adult patients with a confirmed diagnosis of GO were identified through consecutive screening during routine clinical evaluations over the study period. From this pool, eligible patients were allocated to one of three predefined groups based on IOP status and the presence of glaucomatous optic neuropathy: (1) GO with normal IOP (Group A), (2) GO with ocular hypertension (Group B), and (3) GO with clinically diagnosed glaucoma (Group C, primary open angle glaucoma). Recruitment within each group continued until the minimum sample size established by the a priori power calculation (53 patients per group) was reached, with a final sample of 55 patients per group (Figure 1). As this was a quota-based design, the equal group distribution does not reflect the natural prevalence of these conditions in the GO population.

Exclusion criteria included previous intraocular surgery (except uncomplicated cataract surgery), other ocular diseases affecting the optic nerve or retina (diabetic retinopathy, age-related macular degeneration), dysthyroid optic neuropathy, history of optic neuritis, significant refractive error (>±6 diopters), and poor-quality imaging or unreliable visual field tests.

The study adhered to the tenets of the Declaration of Helsinki and was approved by the local Ethics Committee. Written informed consent was obtained from all participants.

### 2.2. Clinical and Ophthalmologic Assessment

All patients underwent a comprehensive ophthalmologic examination, including measurement of best-corrected visual acuity (BCVA) using a Snellen chart (converted to decimal values), slit-lamp biomicroscopy, fundus examination, and Goldmann applanation tonometry for IOP measurement. Intraocular pressure was measured in primary gaze position, and, where clinically indicated, additional measurements were obtained in upgaze to account for extraocular muscle restriction associated with GO. Demographic and clinical data, including age, sex, body mass index (BMI), smoking status, systemic comorbidities (diabetes mellitus, hypertension), stress exposure, and family history, were recorded.

### 2.3. Definition of Ocular Hypertension and Glaucoma

The diagnosis of ocular hypertension and glaucoma in our study population was standardized according to the definitions proposed by the European Glaucoma Society (EGS), 5th Edition [23]. OHT was defined as a consistent elevation of IOP above the statistically normal range (>21 mmHg), in eyes with an open anterior chamber angle and no detectable optic nerve, retinal nerve fiber layer, or visual field damage. Glaucoma was diagnosed in eyes with a normal, open anterior chamber angle showing glaucomatous optic nerve/retinal nerve fiber layer loss with corresponding, reproducible visual field defects, in the absence of an identifiable secondary cause. Visual field testing was not performed in all patients presenting with isolated IOP elevation when no other optic disc or RNFL signs suggestive of glaucoma were present. These criteria were applied uniformly to classify patients into the OHT, glaucoma, and non-glaucomatous groups included in this study.

### 2.4. Optical Coherence Tomography Assessment

Structural retinal parameters were evaluated using spectral-domain optical coherence tomography (SD-OCT) (Cirrus HD-OCT, Carl Zeiss Meditec, Inc., Dublin, CA, USA). The following parameters were recorded: retinal nerve fiber layer (RNFL) thickness, ganglion cell layer plus inner plexiform layer (GCL+IPL) thickness, optic disc area, rim area, cup-to-disc ratio (average and vertical), and cup volume.

All OCT scans were reviewed for quality, and only images with adequate signal strength and without motion artifacts were included in the analysis.

### 2.5. Visual Field Testing

Functional assessment was performed using standard automated perimetry (Humphrey Field Analyzer, Carl Zeiss Meditec, Inc., Dublin, CA, USA), 24-2 SITA Standard. The main parameters analyzed were mean deviation (MD) and pattern standard deviation (PSD).

Visual field tests were considered reliable if fixation losses were <20% and false-positive and false-negative rates were <15%.

### 2.6. Sample Size Calculation

Assuming a one-way ANOVA design with three groups, a medium effect size (Cohen’s f = 0.25), an alpha level of 0.05, and a statistical power of 80%, the minimum required sample size was estimated at 159 participants (approximately 53 per group).

### 2.7. Statistical Analysis

Continuous variables were expressed as mean ± standard deviation, while categorical variables were presented as frequencies and percentages. Between-group comparisons were performed using one-way analysis of variance (ANOVA) for continuous variables and the chi-square test for categorical variables. Where the omnibus test was significant, pairwise comparisons were performed using Tukey’s honestly significant difference test for continuous variables and Fisher’s exact test with Bonferroni correction for categorical variables (Supplementary Table S1).

To identify factors independently associated with glaucoma, a multivariable logistic regression analysis was conducted, restricted to patients with GO and elevated IOP and those with established glaucoma. Two models were constructed: a clinical model including age, intraocular pressure, best-corrected visual acuity, smoking status, and diabetes mellitus, and a combined model that additionally incorporated structural retinal parameters (GCL+IPL thickness). GCL+IPL thickness was selected as the structural variable because it reflects macular ganglion cell integrity independently of optic disc morphology; RNFL, cup-to-disc ratio, rim area, and cup volume were not included, as these parameters also contributed to the structural criteria used to establish the glaucoma diagnosis, and their inclusion would have introduced circularity. Visual field indices (mean deviation, pattern standard deviation) were also not included, as testing was not performed in all patients and the resulting incomplete data would have restricted the regression sample further. Central corneal thickness was measured and compared descriptively across groups but was not included in the regression models. With the sample size available for the multivariable models, adding CCT alongside the existing variables produced unstable estimates for other model terms, indicating the model was overparameterized relative to the available sample; in addition, CCT influences applanation tonometry readings, risking conflation of a genuine risk factor with a measurement property of the IOP variable itself.

Given the presence of correlated variables and the potential for quasi-complete separation, a penalized logistic regression approach was applied to obtain stable estimates.

Model discrimination was assessed using receiver operating characteristic (ROC) curve analysis, and the area under the curve (AUC) was calculated. Internal validation was performed using bootstrap resampling to obtain optimism-corrected estimates of model performance. Model calibration was assessed using the calibration slope and calibration-in-the-large obtained by regressing observed outcomes on the linear predictor of each fitted model; this approach was preferred over the Hosmer–Lemeshow test, which is unstable under arbitrary binning in samples of this size. Spearman rank correlations were computed between OCT-derived structural parameters (RNFL thickness, GCL+IPL thickness, average and vertical C/D ratio, rim area, and cup volume) and visual field indices (mean deviation and pattern standard deviation) in Group C. Results are reported as Spearman rho coefficients with corresponding *p*-values.

A *p*-value < 0.05 was considered statistically significant. Statistical analyses were performed using IBM SPSS Statistics (version 29.0; IBM Corp., Armonk, NY, USA); penalized logistic regression was performed using the FIRTH LOGISTIC extension.

## 3. Results

### 3.1. Baseline Demographic, Structural, and Functional Characteristics

A total of 165 patients with Graves’ orbitopathy were included in the final analysis, distributed equally among the three predefined groups (55 with GO and normal IOP, 55 with GO and ocular hypertension, and 55 with GO and clinically diagnosed glaucoma), consistent with the target sample size established in the a priori power calculation. Baseline demographic and clinical characteristics of the three groups are summarized in Table 1.

Age, BMI, gender, hypertension, stress, and family history did not differ significantly among the three groups (all *p* > 0.05). IOP increased progressively across groups, and BCVA declined progressively, as expected. Smoking prevalence and diabetes mellitus also differed significantly across groups, though not in a strictly progressive pattern. Structural optic nerve head and macular parameters obtained by optical coherence tomography are presented in Table 2. Central corneal thickness differed significantly across groups but did not follow a monotonic pattern with disease severity, with Group B showing the greatest thickness of the three groups rather than an intermediate value between Group A and Group C.

Rim area and disc area did not differ significantly among the three groups (*p* = 0.588 and *p* = 0.924, respectively). In contrast, RNFL thickness, average and vertical cup-to-disc ratios, cup volume, and GCL+IPL thickness all differed significantly across groups, though the pattern of differences was not uniformly progressive across the three groups. 

Table 3 exhibits functional visual field parameters. These data represent subset analyses only, as perimetry was available for 23 of 55 patients in Group A, 19 of 55 in Group B, and all 55 patients in Group C, and should be interpreted accordingly.

Within the glaucoma group, Spearman correlations between structural OCT parameters and mean deviation revealed significant associations for optic disc parameters—rim area (rho = −0.795, *p* < 0.001), cup volume (rho = −0.795, *p* < 0.001), average C/D ratio (rho = −0.800, *p* < 0.001), and vertical C/D ratio (rho = −0.797, *p* < 0.001), while GCL+IPL thickness did not correlate significantly with mean deviation (rho = −0.057, *p* = 0.81). RNFL thickness showed a non-significant trend (rho = −0.384, *p* = 0.094).

Both mean deviation and pattern standard deviation differed significantly across the three groups, with the greatest functional impairment observed in the Group C, as expected.

Post-hoc comparisons showed that most structural and functional parameters (RNFL, average cup-to-disc ratio, cup volume, GCL+IPL thickness, MD, and PSD) differed significantly between the Group C and both other groups, but not between Group A and Group B, therefore indicating these parameters primarily distinguish established glaucoma rather than ocular hypertension alone. Vertical cup-to-disc ratio showed the reverse pattern, differing significantly between Group A and Group B, and Group A and Group C, but not between Group B and Group C, (Supplementary Table S1).

### 3.2. Factors Associated with Glaucoma Status: Multivariable Logistic Regression and Diagnostic Performance

To identify factors independently associated with glaucoma in patients with GO, a multivariable logistic regression analysis was performed. The analysis was restricted to patients with GO and elevated intraocular pressure and those with established glaucoma in order to evaluate which factors distinguish established glaucoma from ocular hypertension within this at-risk subgroup. Patients with normal IOP were not included in this analysis. Two models were constructed: a clinical model which included age, intraocular pressure, best-corrected visual acuity, smoking status, and diabetes mellitus (Table 4), and a combined model (Table 5) that additionally incorporated a structural retinal parameter (average ganglion cell layer plus inner plexiform layer thickness). Given the presence of correlated variables and the risk of quasi-complete separation, a penalized logistic regression approach was applied to obtain stable and unbiased estimates.

In the clinical model, intraocular pressure was independently associated with glaucoma, with each 1 mmHg increase corresponding to a 44% increase in the odds of glaucoma. Best-corrected visual acuity showed a significant inverse association. Diabetes mellitus was independently associated with increased odds of glaucoma (OR 3.10). Smoking was independently associated with reduced odds of glaucoma in this comparison (OR 0.25); given the absence of an established biological mechanism for a protective effect and the binary nature of the smoking variable, which does not capture intensity or duration, this finding should be interpreted with caution and requires replication rather than causal interpretation. Age was not significantly associated with glaucoma in this model.

After inclusion of the optical coherence tomography parameter, intraocular pressure remained the factor most strongly and independently associated with glaucoma status, with a slightly increased effect size compared to the clinical model. GCL+IPL thickness was independently associated with glaucoma (OR 0.90, *p* = 0.036), with thinner GCL+IPL associated with increased odds of glaucoma even after adjustment for clinical variables and the confounders identified in Table 1. Best-corrected visual acuity showed a weaker, non-significant association in this model (*p* = 0.089). Diabetes mellitus and smoking retained the associations observed in the clinical model. Age remained non-significant in the combined model.

**Figure 2:**
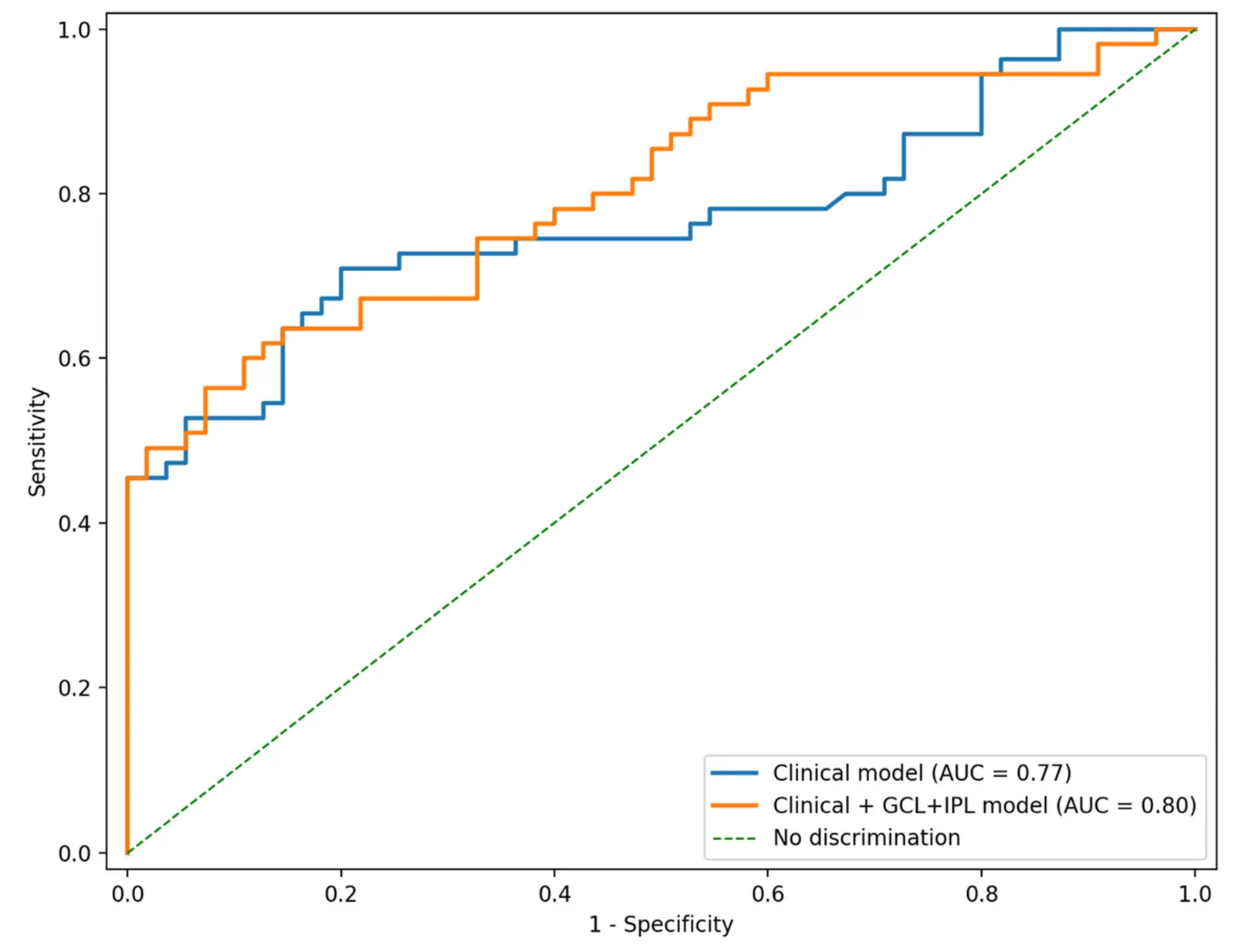
Receiver operating characteristic curves comparing the discriminative ability of the clinical model and the combined clinical and OCT model for glaucoma in patients with Graves’ orbitopathy. AUC values shown are apparent (in-sample) estimates; optimism-corrected estimates are reported in the text.

The addition of structural parameters resulted in an improvement in overall model discrimination, as reflected by the increase in the apparent area under the receiver operating characteristic curve from 0.77 in the clinical model to 0.80 in the combined model (Figure 2). Therefore, retinal structural assessment provided incremental diagnostic value beyond clinical variables alone.

**Figure 3:**
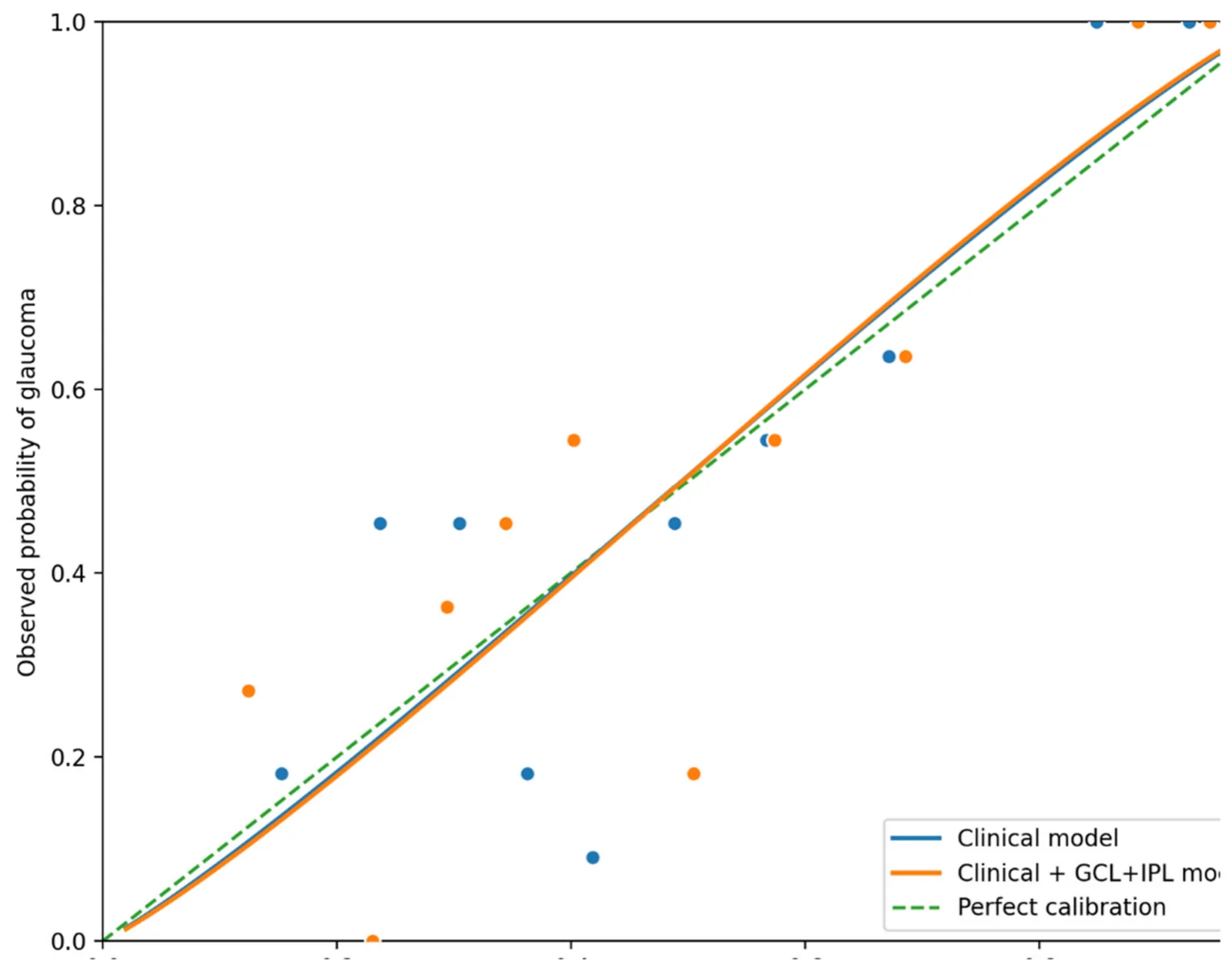
Calibration plots for the clinical model and the combined clinical and OCT model, showing observed versus estimated probability of glaucoma in the 110-patient logistic regression subsample (Groups B and C). Solid lines show the fitted recalibration curve for each model; points show observed versus mean estimated probability within deciles of predicted risk. The dashed diagonal represents perfect calibration.

Receiver operating characteristic curve analysis with bootstrap resampling demonstrated that the clinical model showed moderate discriminative ability for identifying glaucoma, with an optimism-corrected area under the curve of 0.73 (range 0.65–0.82). After inclusion of structural retinal parameters, specifically ganglion cell layer plus inner plexiform layer thickness, the discriminative performance improved, with an optimism-corrected AUC of 0.76 (range 0.69–0.84). Both models were well calibrated (Figure 3): the calibration slope was 1.09 (intercept 0.02) for the clinical model and 1.11 (intercept 0.02) for the combined model.

Intraocular pressure remained the most consistent independent predictor of glaucoma across models. GCL+IPL thickness was independently associated with glaucoma after adjustment for clinical variables and confounders.

## 4. Discussion

In the present study of patients with Graves’ orbitopathy stratified by IOP and glaucoma status, most structural and functional parameters distinguished established glaucoma from ocular hypertension, but not ocular hypertension from normal IOP; detectable retinal damage tracked the presence of glaucoma itself, rather than IOP elevation alone. In multivariable analysis, IOP showed the strongest and most consistent independent association with glaucoma status (OR per 1 mmHg increase: 1.44 in the clinical model, 1.61 after adjustment for structural parameters and confounders), outperforming structural OCT assessment; GCL+IPL thickness was independently associated with glaucoma after adjustment (OR 0.90), improving model discrimination (optimism-corrected AUC from 0.73 to 0.76).

Elevated IOP is a well-established risk factor for glaucoma in the general population, and although several studies confirm a link between GO and ocular hypertension, the reported incidence of ocular hypertension and open-angle glaucoma across GO cohorts varies widely and remains debated [24,25]. Additionally, ocular hypertension in GO does not always progress to glaucoma. A large retrospective cohort of 1128 GO patients found ocular hypertension in 6.8% but glaucoma in only 1.6%, and only 1 of 77 ocular hypertension patients progressed to glaucoma during follow-up. The study also listed thin central corneal thickness among established glaucoma risk factors [24]. A population-based study found that thyroid disease was linked to open-angle glaucoma mainly through current thyroxine use (OR 2.1) and prior thyroid surgery (OR 2.5), rather than thyroid disease history alone, with a possible thyroid-axis contribution to glaucoma risk distinct from the orbital mechanical effects of GO [26].

Proposed mechanisms of IOP elevation in GO include elevated episcleral venous pressure, glycosaminoglycan deposition within the trabecular meshwork, restrictive myopathy, and steroid-induced glaucoma, though the relative contribution of each remains unclear [27,28,29].

OCT has revolutionized the research of retinal and choroidal involvement in GO by allowing thorough, non-invasive examination of these layers, providing knowledge of the vascular and inflammatory processes underlying the disease [30,31]. Even so, differences in imaging protocols, patient populations, and disease severity across studies continue to produce inconsistent results [32]. Among the OCT parameters, RNFL and GCL+IPL thickness are of particular relevance to IOP-related damage, as they directly reflect axonal and ganglion cell integrity at the optic nerve and macula rather than orbital inflammatory change alone [33,34]. RNFL and GCL+IPL thinning has been reported specifically in patients with GO, and not in those with Graves’ disease without orbitopathy, compared to healthy controls [35]. Within GO, this thinning did not differ significantly between patients with and without dysthyroid optic neuropathy [36]. One study found no significant difference in macular ganglion cell complex thickness across GO severity groups.

Bagatin et al. [19] compared GO and GO+IOP patients against an external cohort of primary open-angle glaucoma, using sector-based Octopus Polar Analysis correlated with clock-hour OCT sectors, and found RNFL thinning, rim thinning, and increased cup-to-disc ratio already present at the GO+IOP stage relative to GO, with the most pronounced changes in the POAG group. Their multivariable model targeted elevated IOP itself as the outcome.

A systematic review of 20 studies similarly found inconsistent RNFL and GCL/IPL differences between non-DON GO patients and controls, with reliable thinning only once compressive optic neuropathy was present [37] A study comparing GO patients with and without elevated IOP found lower RNFL and GCL-IPL thickness in the raised-IOP group across every sector measured, but none of these differences reached significance [38]. One study found a difference limited to the temporal quadrant that didn’t show up in the global average [39].

Although macular GCL+IPL thinning is classically associated with more advanced glaucomatous damage [40,41], growing evidence suggests that the macular vulnerability zone shows detectable structural loss even in early to moderate stages of the disease [42], with studies showing macular GCL+IPL thickness performing comparably to peripapillary RNFL in detecting early glaucomatous damage [43]. In this study, GCL+IPL thickness was independently associated with glaucoma status in the combined model, which may reflect its sensitivity to the cumulative ganglion cell loss that characterizes established glaucoma rather than early functional change. The exploratory structure-function analysis within the glaucoma group suggested that optic disc parameters tracked functional loss more closely than GCL+IPL.

The unexpected inverse association between smoking and glaucoma warrants closer scrutiny. Smoking status was ascertained from existing clinical records and not through biochemical verification and therefore relied on patient self-report at the time of consultation, which cannot be assumed to be fully reliable; despite this limitation, smoking status was retained as a covariate because it differed significantly across groups and its omission risked residual confounding. Moreover, smoking prevalence itself followed a non-monotonic pattern across groups (47.27% in Group A, 83.64% in Group B, and 58.18% in Group C), and because the multivariable models were restricted to Groups B and C, part of the apparent inverse association between smoking and glaucoma may reflect the unusually high smoking prevalence in the GO+IOP comparison group rather than a genuine protective effect.

The two-model comparison provides a quantitative estimate of the diagnostic value added by structural OCT assessment beyond variables already collected in routine clinical care. GCL+IPL thickness reached independent significance once smoking and diabetes mellitus were accounted for. These data indicate that periodic IOP and visual acuity monitoring remain critical to first-line glaucoma risk assessment in GO, with structural OCT examination providing extra, supplementary diagnostic value. The incomplete visual field coverage also reflects a practical challenge inherent to retrospective designs: perimetry is usually used when structural findings raise concern, meaning the tested subset is not a random sample of the group. Fully characterizing the functional dimension of IOP-related damage in GO would require prospective studies with systematic perimetry across all IOP strata, independent of disc or RNFL appearance.

### Strengths and Limitations

Several limitations of this study should be considered when interpreting the findings. First, its retrospective, cross-sectional design precludes causal or temporal inference. Second, the study was conducted at a single center, which may limit generalizability. Third, visual field comparisons represent subset analyses rather than full group comparisons, as perimetry was not performed uniformly. Testing was indicated when optic disc morphology or RNFL findings raised clinical concern, not in patients with isolated IOP elevation and no structural signs of damage. Patients who underwent perimetry in Groups A and B are therefore a selected subgroup with a higher chance of structural change, which may inflate the apparent functional differences between groups. Fourth, only GCL+IPL thickness was entered as a structural variable in the multivariable models; RNFL, cup-to-disc ratios, rim area, and cup volume were deliberately excluded because they also contributed to the structural criteria used to diagnose glaucoma, and their inclusion would have introduced circularity. Fifth, OCTA parameters, which may offer additional diagnostic value in GO, were not assessed. Sixth, disease duration, GO-directed treatment history, and inter-observer variability in OCT and perimetry interpretation were not recorded and may have confounded the observed group differences. CCT followed a non-monotonic pattern across groups; since Goldmann applanation tonometry tends to over-read IOP in thicker corneas and under-read it in thinner ones, the IOP values recorded in these two groups may not fully reflect their true biological pressures, and the true separation between ocular hypertension and glaucoma could be larger than the measured difference. Finally, as all three groups in this study had GO, the absence of a non-GO control group means it cannot be excluded that some degree of structural thinning was already present in the normal-IOP group relative to disease-free eyes.

## 5. Conclusions

In this cross-sectional study of patients with Graves’ orbitopathy, intraocular pressure showed the strongest and most consistent independent association with glaucoma status across clinical and combined models. Diabetes mellitus was independently associated with increased odds of glaucoma r in both models, while smoking showed an unexpected inverse association warranting further investigation. GCL+IPL thickness was independently associated with glaucoma status once these confounders were accounted for, improving model discrimination. These findings suggest that regular IOP and visual acuity monitoring may represent a reasonable first-line approach in the follow-up of GO patients, with structural OCT assessment as a useful adjunct. Larger, prospective, multi-center studies are needed to determine whether these associations translate into clinically useful tools.

## Figures and Tables

**Table 1:** Demographic and clinical characteristics of patients with Graves’ orbitopathy.

Variable	Group A	Group B	Group C	*p*-Value
Age (years)	46.76 ± 9.47	47.44 ± 9.99	47.33 ± 10.04	0.929
BMI (kg/m^2^)	27.79 ± 4.13	27.80 ± 4.27	27.17 ± 3.80	0.650
BCVA	0.81 ± 0.13	0.77 ± 0.15	0.65 ± 0.26	<0.001
IOP (mmHg)	17.24 ± 2.31	24.04 ± 1.48	25.16 ± 2.35	<0.001
CCT (µm)	549.2 ± 24.8	589.7 ± 29.4	526.2 ± 27.0	<0.001
Gender, female, n (%)	34 (61.82%)	35 (63.64%)	37 (67.27%)	0.831
Smokers, n (%)	26 (47.27%)	46 (83.64%)	32 (58.18%)	<0.001
Diabetes mellitus, n (%)	15 (27.27%)	7 (12.73%)	19 (34.55%)	0.026
Hypertension, n (%)	22 (40.00%)	31 (56.36%)	23 (41.82%)	0.169
Stress, n (%)	42 (76.36%)	44 (80.00%)	44 (80.00%)	0.865
Family history, n (%)	12 (21.82%)	7 (12.73%)	5 (9.09%)	0.149

GO—Graves’ orbitopathy; IOP—intraocular pressure; BMI—body mass index; BCVA—best-corrected visual acuity; CCT—central corneal thickness.

**Table 2:** Optical coherence tomography–derived optic nerve head and macular parameters across the three study groups.

Variable	Group A	Group B	Group C	*p*-Value
Rim area (mm^2^)	1.69 ± 0.36	1.61 ± 0.36	1.64 ± 0.38	0.588
Disc area (mm^2^)	1.96 ± 0.48	1.94 ± 0.42	1.92 ± 0.56	0.924
RNFL (µm)	90.05 ± 10.91	87.31 ± 11.00	78.76 ± 12.82	<0.001
Average C/D ratio	0.36 ± 0.14	0.40 ± 0.18	0.50 ± 0.20	<0.001
Vertical C/D ratio	0.33 ± 0.13	0.46 ± 0.18	0.52 ± 0.22	<0.001
Cup volume (mm^3^)	0.33 ± 0.14	0.38 ± 0.20	0.72 ± 0.36	<0.001
GCL+IPL thickness (µm)	72.27 ± 5.65	70.07 ± 4.79	69.05 ± 6.04	0.009

C/D—cup-to-disc ratio; GCL+IPL—ganglion cell layer plus inner plexiform layer.

**Table 3:** Visual field parameters across the three study groups.

Variable	Group A (n = 23)	Group B (n = 19)	Group C (n = 55)	*p*-Value
Mean deviation (dB)	−1.23 ± 0.78	−1.34 ± 0.86	−5.74 ± 2.67	<0.001
Pattern standard deviation	0.77 ± 0.51	1.02 ± 0.61	5.34 ± 1.50	<0.001

MD—mean deviation; PSD—pattern standard deviation reported as an unsigned value per standard Humphrey Field Analyzer convention.

**Table 4:** Multivariable logistic regression (clinical model): factors associated with glaucoma status among patients with Graves’ orbitopathy and elevated intraocular pressure or established glaucoma.

Variable	OR	95% CI	*p*-Value
Age	1.02	0.97–1.06	0.450
IOP	1.44	1.13–1.83	0.003
BCVA	0.07	0.01–0.79	0.032
Smoking status (yes)	0.25	0.09–0.70	0.008
DM	3.10	1.02–9.40	0.045

OR—odds ratio; CI—confidence interval; DM-diabetes mellitus.

**Table 5:** Multivariable logistic regression (combined model, incorporating structural OCT parameters): factors associated with glaucoma status among patients with Graves’ orbitopathy and elevated intraocular pressure or established glaucoma.

Variable	OR	95% CI	*p*-Value
Age	1.02	0.98–1.07	0.346
IOP	1.61	1.23–2.12	0.001
BCVA	0.11	0.01–1.40	0.089
GCL+IPL	0.90	0.82–0.99	0.036
Smoking status (yes)	0.21	0.07–0.63	0.005
DM	3.67	1.13–11.91	0.030

OR—odds ratio; CI—confidence interval; GCL+IPL—ganglion cell layer plus inner plexiform layer.

## Data Availability

The data presented in this study are available on request from the corresponding author.

## Supplementary Materials

The following supporting information can be downloaded at: https://www.mdpi.com/article/10.3390/jcm15197741/s1, Table S1: Post-hoc pairwise comparisons of demographic, clinical, structural, and functional parameters across the three groups.

## Abbreviations

The following abbreviations are used in this manuscript:

GO	Graves’ orbitopathy
GD	Graves’ disease
TRAb	thyrotropin receptor antibody
DON	dysthyroid optic neuropathy
IOP	intraocular pressure
BMI	body mass index
BCVA	best-corrected visual acuity
DM	diabetes mellitus
OCT	optical coherence tomography
OCTA	optical coherence tomography angiography
SD-OCT	spectral-domain optical coherence tomography
RNFL	retinal nerve fiber layer
GCL+IPL	ganglion cell layer plus inner plexiform layer
C/D	cup-to-disc (ratio)
MD	mean deviation
PSD	pattern standard deviation
ROC	receiver operating characteristic
AUC	area under the curve
OR	odds ratio
CI	confidence interval
