# Supplementary material for: Ocular Hypertension and Glaucoma in Graves’ Orbitopathy: A Single-Center Retrospective Cross-Sectional Study
Source: J Clin Med. 2026 Oct 8;15(19):7741. doi: 10.3390/jcm15197741 (PMC13666383; doi:10.3390/jcm15197741)
Supplement: Supplementary file 1 [file jcm-15-07741-s001.zip › jcm-4594656-supplementary.pdf]

Table S1. Post-hoc pairwise comparisons of demographic, clinical, structural, and functional parameters across the group A, group B, and group C. Continuous variables were compared using Tukey's honestly significant difference (HSD) test following one-way ANOVA; categorical variables were compared using Fisher's exact test with Bonferroni correction for three pairwise comparisons. IOP — intraocular pressure; BMI — body mass index; BCVA — best-corrected visual acuity; C/D — cup-to-disc ratio; GCL+IPL — ganglion cell layer plus inner plexiform layer. Bold p-values indicate statistical significance ($p < 0.05$).

Demographic and clinical parameters (Tukey HSD)

Variable	Comparison	Mean difference	Adjusted p-value
Age (yrs)	group A vs group B	0.67	0.9315
	group A vs group C	0.56	0.9514
	group B vs group C	-0.11	0.9981
BMI (kg/m²)	group A vs group B	0.01	0.9999
	group A vs group C	-0.62	0.7045
	group B vs group C	-0.63	0.6973
BCVA	group A vs group B	-0.04	0.4723
	group A vs group C	-0.16	**0.0001**
	group B vs group C	-0.12	**0.0041**
IOP (mmHg)	group A vs group B	6.80	**<0.0001**
	group A vs group C	7.93	**<0.0001**
	group B vs group C	1.13	**0.0141**

Optical coherence tomography parameters (Tukey HSD)

Variable	Comparison	Mean difference	Adjusted p-value
Rim area (mm²)	group A vs group B	-0.07	0.5632
	group A vs group C	-0.04	0.8018
	group B vs group C	0.03	0.9198
Disc area (mm²)	group A vs group B	-0.02	0.9798
	group A vs group C	-0.04	0.9164
	group B vs group C	-0.02	0.9769
RNFL (μm)	group A vs group B	-2.75	0.4314
	group A vs group C	-11.29	**<0.0001**
	group B vs group C	-8.55	**0.0005**
Average C/D ratio	group A vs group B	0.04	0.4985
	group A vs group C	0.14	**0.0002**
	group B vs group C	0.10	**0.0098**
Vertical C/D ratio	group A vs group B	0.13	**0.0007**
	group A vs group C	0.19	**<0.0001**
	group B vs group C	0.06	0.2082
Cup volume (mm³)	group A vs group B	0.06	0.4661
	group A vs group C	0.40	**<0.0001**
	group B vs group C	0.34	**<0.0001**
GCL+IPL thickness (μm)	group A vs group B	-2.20	0.0948
	group A vs group C	-3.22	**0.0073**
	group B vs group C	-1.02	0.5982

Visual field parameters (Tukey HSD)

Variable	Comparison	Mean difference	Adjusted p-value
Mean deviation (dB)	group A vs group B	-0.10	0.9771

Variable	Comparison	Mean difference	Adjusted p-value
	group A vs group C	-4.51	**<0.0001**
	group B vs group C	-4.41	**<0.0001**
Pattern standard deviation (dB)	group A vs group B	0.25	0.6566
	group A vs group C	4.57	**<0.0001**
	group B vs group C	4.32	**<0.0001**

Categorical risk factors (Fisher's exact test, Bonferroni-corrected)

Variable	Comparison		Adjusted p-value
Female sex	group A vs group B	—	1.0000
	group A vs group C	—	1.0000
	group B vs group C	—	1.0000
Smokers	group A vs group B	—	**0.0003**
	group A vs group C	—	1.0000
	group B vs group C	—	**0.0176**
Diabetes mellitus	group A vs group B	—	0.2813
	group A vs group C	—	1.0000
	group B vs group C	—	**0.0379**
Hypertension	group A vs group B	—	0.3795
	group A vs group C	—	1.0000
	group B vs group C	—	0.5448
Stress	group A vs group B	—	1.0000
	group A vs group C	—	1.0000
	group B vs group C	—	1.0000
Family history	group A vs group B	—	0.9394
	group A vs group C	—	0.3349
	group B vs group C	—	1.0000
